# Dysfunctions of multiscale dynamic brain functional networks in subjective cognitive decline

**DOI:** 10.1093/braincomms/fcae010

**Published:** 2024-01-16

**Authors:** Mianxin Liu, Qi Huang, Lin Huang, Shuhua Ren, Liang Cui, Han Zhang, Yihui Guan, Qihao Guo, Fang Xie, Dinggang Shen

**Affiliations:** Shanghai Artificial Intelligence Laboratory, Shanghai 200232, China; School of Biomedical Engineering, State Key Laboratory of Advanced Medical Materials and Devices, ShanghaiTech University, Shanghai 201210, China; Department of Nuclear Medicine & PET Center, Huashan Hospital, Fudan University, Shanghai 200040, China; Department of Gerontology, Shanghai Jiao Tong University Affiliated Sixth People’s Hospital, Shanghai 200233, China; Department of Nuclear Medicine & PET Center, Huashan Hospital, Fudan University, Shanghai 200040, China; Department of Gerontology, Shanghai Jiao Tong University Affiliated Sixth People’s Hospital, Shanghai 200233, China; School of Biomedical Engineering, State Key Laboratory of Advanced Medical Materials and Devices, ShanghaiTech University, Shanghai 201210, China; Department of Nuclear Medicine & PET Center, Huashan Hospital, Fudan University, Shanghai 200040, China; Department of Gerontology, Shanghai Jiao Tong University Affiliated Sixth People’s Hospital, Shanghai 200233, China; Department of Nuclear Medicine & PET Center, Huashan Hospital, Fudan University, Shanghai 200040, China; School of Biomedical Engineering, State Key Laboratory of Advanced Medical Materials and Devices, ShanghaiTech University, Shanghai 201210, China; Shanghai United Imaging Intelligence Co., Ltd., Shanghai 200230, China; Shanghai Clinical Research and Trial Center, Shanghai, 201210, China

**Keywords:** dynamic functional connectivity, functional magnetic resonance imaging, subjective cognitive decline, graph convolutional network, Alzheimer’s disease

## Abstract

Subjective cognitive decline is potentially the earliest symptom of Alzheimer's disease, whose objective neurological basis remains elusive. To explore the potential biomarkers for subjective cognitive decline, we developed a novel deep learning method based on multiscale dynamical brain functional networks to identify subjective cognitive declines. We retrospectively constructed an internal data set (with 112 subjective cognitive decline and 64 healthy control subjects) to develop and internally validate the deep learning model. Conventional deep learning methods based on static and dynamic brain functional networks are compared. After the model is established, we prospectively collect an external data set (26 subjective cognitive decline and 12 healthy control subjects) for testing. Meanwhile, our method provides monitoring of the transitions between normal and abnormal (subjective cognitive decline–related) dynamical functional network states. The features of abnormal dynamical functional network states are quantified by network and variability metrics and associated with individual cognitions. Our method achieves an area under the receiver operating characteristic curve of 0.807 ± 0.046 in the internal validation data set and of 0.707 (*P* = 0.007) in the external testing data set, which shows improvements compared to conventional methods. The method further suggests that, at the local level, the abnormal dynamical functional network states are characterized by decreased connectivity strength and increased connectivity variability at different spatial scales. At the network level, the abnormal states are featured by scale-specifically altered modularity and all-scale decreased efficiency. Low tendencies to stay in abnormal states and high state transition variabilities are significantly associated with high general, language and executive functions. Overall, our work supports the deficits in multiscale brain dynamical functional networks detected by the deep learning method as reliable and meaningful neural alternation underpinning subjective cognitive decline.

## Teaser

Liu *et al*. developed a deep learning method based on multiscale dynamical brain functional networks to identify subjective cognitive decline with high accuracy and reliability. They demonstrated that the neurological deficits underlying subjective cognitive decline manifest as the alternations of link-level, network-level and transition properties of dynamical brain functional networks.

## Graphical Abstract

**Graphical Abstract:**
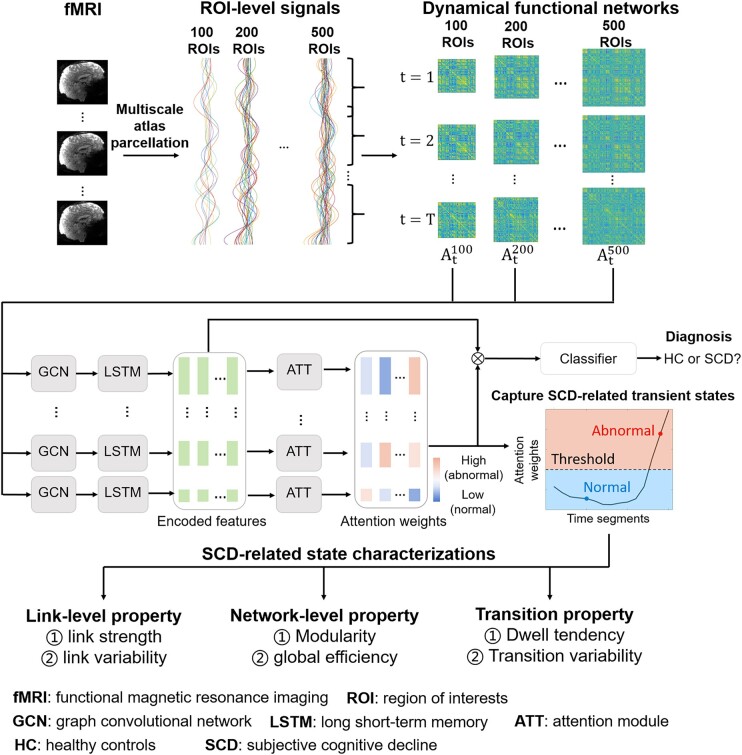

## Introduction

Subjective cognitive decline (SCD) refers to a self-reported decline of cognitive functions (not restricted in memory) and is regarded as a symptom or risk factor of incipient dementia.^1,2^ Previous literature prevalently discussed the potential role of SCD as a starting stage before objective mild cognitive impairment and dementia in Alzheimer's disease continuum.^1-4^ However, the clinical significance of SCD has been controversial, largely due to its definition based on subjective perceptions and the lack of a reliable manifestation of neuroabnormality to be targeted. Locating the objective neurological alternations behind the self-perceived cognitive decline can pave the path for establishing SCD as an effective clinical entity to corroborate incipient dementia.^5-7^

Recently, studies using functional MRI (fMRI) demonstrated the deficits in brain information processing in memory and executive functions under the SCD.^7-12^ In task-state fMRI studies for SCDs, group-wise differences in task-related brain activations have been found during memory encoding and retrieval, working memory and other executive functions.^13-16^ In addition, the functional connectivities (FCs) extracted from fMRI under resting states (without explicit cognitive loads) are used to capture the information signalling by measuring the temporal synchronizations between activities from two brain regions. Cohort studies found that SCD can be associated with aberrant FCs in default mode network (DMN),^10,17,18^ saliency network^19^ and hippocampus.^20,21^ Despite these aberrant local connections, characterizations based on graph theory metrics for the subjects under SCD demonstrate decreased network efficiency and increased modularity,^17^ suggesting an interrupted functional integration and segregation in the brain functional network.

More recently, it was widely observed that the architecture of the brain-wide FC network can reconfigure over time,^22^ and thus, a dynamic FC network (dFCN) method can more properly capture the temporal details of the brain information exchanges. Emerging evidence suggests dependencies between altered dFCN properties and SCD. Dong *et al*.^23^ reported increased dFC flexibility in the left inferior frontal gyrus and decreased flexibility in the left inferior temporal gyrus in the SCD population, while higher flexibility in both regions is associated with higher behavioural scores. This evidence implies compensatory processing via flexible re-organizations of the brain networks to maintain normal cognitive functions under insufficient information processing.^24^ In addition, the dFCNs were found to recurrently switch among several typical states, and the SCD status can be correlated to increased dwell time in specific states and less frequent transition among the states.^12,25^

Despite the novelty and success of these latest developments, the investigation of dysfunction in brain functional interactions under SCD remains insufficient due to two major lacks. First, most of the prevalent studies use a single-scale atlas to construct dFCNs. However, the brain is known as a multiscale hierarchical system.^26,27^ It has already been revealed that the multiscale hierarchical organization is crucial for brain information processing, such as facilitating the balance of information integration and segregation^28,29^ and improving the sensitivity and reliability to response to outer stimulus.^30,31^ What's more, mild cognitive impairment and Alzheimer's disease have been shown to induce FC dysfunctions across multiple spatial scales.^32-35^ Therefore, dysfunctions in the multiscale brain information exchanges could underlie SCD as well, which previous single-scale analyses can neglect.

Second, the features of SCD-related dFCN states, i.e. the transient network configurations, need to be more clearly elucidated to understand the deficits in neural processing. Global metrics such as temporal flexibility or variability have been extracted from the dFCNs to predict the SCD,^23,36^ but these studies did not go deep into the characterizations of the SCD-related dFCN states. Another study applied clustering methods to group the dFCN states and applied group comparison methods to reveal and explore the atypical dFCN states *post hoc*.^12,25^ Such analysis framework only investigated within-sample relationships and thus cannot guarantee the generalizability of the detected state and its association with the SCD. Also, the conventional method can only reveal a linear association between the dFCN state changes and the symptoms, but the complex pathology can often induce non-linearity in the brain–behaviour relationships. Tackling the non-linear dependency with more advanced data analysis methods, such as machine learning (especially deep learning), can thus be necessary.^37-41^ So far, there is seldom a study identifying the SCD-related dFCN states and testing the diagnostic powers of the detected dFCN states using advanced deep learning methods and prediction frameworks.

In the presented work, we develop a novel deep learning method to identify SCD and validate the SCD-related deficits shown in brain dFCNs at multiple spatial scales. We build multiscale dFCNs with advanced functional parcellation atlases and detect the SCD-related dFCN states at multiple spatial scales, leveraging deep graph learning methods. Under prediction frameworks, we demonstrate the sensitivity and generalizability of the abnormality in detected states for identifying SCD subjects. In addition, we comprehensively investigate the edge-level and network-level differences between the normal and SCD-related states and the dependencies among the temporal properties of the dFCN state transitions and the clinical psychometrics scores.

## Materials and methods

### Ethics approval and consent to participate

This study is approved by the Institutional Ethical Reviewing Board of Huashan Hospital and Shanghai Jiao Tong University Affiliated Sixth People's Hospital. All subjects (right-handed, aged between 50 and 80 years old) provided written informed consent before any procedures.

### Subjects

The data collection has been registered as ‘ChiCTR2000036842’ and entitled ‘Construction of pre-clinical Ad neuroimaging cohort of ATN system’ (see URL of registry http://www.chictr.org.cn/showproj.aspx?proj=59802). The data collection for the presented study started in January 2021 and ended in October 2023. This study further excludes subjects with low education levels (≤5 years), neurological or psychiatric antecedents, other neurological conditions or diseases that could cause cognitive decline other than Ad spectrum disorders and significant alcohol or drug abuse. A total of 217 subjects, including 76 healthy controls (HCs) and 138 SCD patients, were recruited from communities in Shanghai. The sample size was determined before recruiting the patients based on pre-experiments using group comparison methods (two-sided two-sample *t*-tests) in a small-sample data set. Using the PASS software (https://www.ncss.com/software/pass/), given the alpha = 0.05 and power = 0.80 and the mean and the standard deviations from the small-sample data set, we estimated that roughly 70 samples for HC and SCD groups could be enough to achieve the same effect size as in the pre-experiment. We do admit the discrepancy between the group comparison method and the deep learning method but emphasize that our method design based on the dFCN states from subjects could ease the sample size problem (see below model details). For each subject, we extract 22 dFCN states, which could to some extent be regarded as respective samples being utilized to train shared deep learning modules. In this manner, the model could be robust and suffer less from the overfitting problem. In the deep learning field, this framework is called ‘multiple instance learning’.^42^

The data set contains the subjects from a retrospective internal validation data set (collected before the establishment of the proposed deep learning model, i.e. before February 2023) and a prospective external testing data set (collected after the model is established and fixed, i.e. after February 2023). The internal validation data set includes 176 subjects (64 HCs and 112 SCDs), with controls in gender, age and education level (Table 1). The external testing data set involves 38 subjects (12 HCs and 26 SCDs) without any controls to mimic practical situations.

**Table 1:** The characteristics of the subjects in groups

	Retrospective internal validation			Prospective external testing		
	HC	SCD	*P* ^a^	HC	SCD	*P* ^a^
Gender^b^	25:39	32:80	0.15	7:5	7:19	0.06
Age, years	64.8 ± 9.1	62.8 ± 7.0	0.11	62.9 ± 8.4	61.6 ± 9.1	0.68
Education, y^c^	12.8 ± 3.3	12.2 ± 2.8	0.26	13.5 ± 3.8	12.9 ± 3.2	0.64
MMSE^d^	28.7 ± 1.2	28.2 ± 1.7	0.06	28.3 ± 1.7	28.6 ± 1.1	0.60
MoCA-B^d^	26.7 ± 2.4	26.0 ± 2.6	0.05	25.6 ± 2.0	25.8 ± 3.1	0.85
AVLT-FDR	6.7 ± 1.8	6.4 ± 2.2	0.29	5.9 ± 1.8	6.2 ± 2.3	0.75
AVLT-R	21.1 ± 4.6	21.5 ± 3.6	0.46	22.4 ± 1.2	22.1 ± 1.9	0.63
AFT	17.5 ± 3.1	17.2 ± 5.1	0.68	19.9 ± 3.3	18.0 ± 6.2	0.31
BNT	25.0 ± 3.9	24.0 ± 4.8	0.16	25.4 ± 2.6	25.2 ± 3.7	0.83
STT-A, s^e^	45.4 ± 13.9	44.5 ± 14.1	0.68	40.5 ± 10.0	43.0 ± 13.5	0.57
STT-B, s^e^	114.8 ± 38.2	109.8 ± 34.1	0.38	117.1 ± 26.4	125.8 ± 40.3	0.50
HAMD^d^	3.5 ± 4.2	5.3 ± 4.1	0.008	2.4 ± 2.9	3.5 ± 4.5	0.65
HAMA^d^	4.4 ± 4.7	6.8 ± 5.6	0.01	4.7 ± 4.6	5.5 ± 4.9	0.48
IDS-SR^d^	9.1 ± 6.8	13.3 ± 8.8	0.001	7.8 ± 7.8	12.4 ± 10.0	0.11

^a^ *P*-values are not corrected. ^b^Formatted as ‘male:female’. Tested by $\chi^{2}$ test. ^c^Education years. ^d^Tested by Whitney–Mann's U-test. ^e^Measured as the time cost (in s) to finish the test (fast completion indicates high cognitive abilities).

### Neuropsychology

Systematic Chinese background–based neuropsychological assessments are conducted by trained neuropsychologists in Shanghai Jiao Tong University Affiliated Sixth People's Hospital. Firstly, the global cognitions are examined by Mini-Mental State Examination (MMSE) and Montreal Cognitive Assessment-Basic (MoCA-B). Besides, six neuropsychological scores are assessed to cover memory, language and executive cognitive domains: auditory verbal learning test (AVLT) 30-min long-delayed free recall (AVLT-LDR) and AVLT-recognition (AVLT-R) for memory function domain; animal fluency test (AFT, total score) and 30-item Boston Naming Test (BNT, total score) for language function domain; and shape trail test (STT), parts A and B (STT-A and STT-B, time to completion), for executive function domain.^43-47^ All measurements indicate higher cognitive ability with higher scores, except STT-A and STT-B. In addition, psychological assessments for anxiety and depression are tested, including the Hamilton Anxiety Rating Scale (HAMA), Hamilton Depression Rating Scale (HAMD) and 30-item versions of the Inventory of Depressive Symptomatology, Self-Report (IDS-SR).

After the exclusion of mild cognitive impairment and dementia, the cognitively unimpaired subjects are further classified as SCD based on the research criteria proposed by previous literature.^2,48^ Two features are required to meet the criteria: (i) subjective decline in any cognitive domain and (ii) concerns associated with SCD. The current cognitively unimpaired individuals who do not exhibit objective cognitive impairments and SCD are identified as HC subjects.

### MRI

High-resolution 3D MRI is performed on a 3T MRI scanner (Prisma 3.0T, Siemens, Erlangen, Germany). MRI T_1_ images are acquired with a 3D magnetization-prepared rapid gradient echo sequence with the following parameters: repetition time = 3000 ms, echo time = 2.56 ms, flip angle = 7°, acquisition matrix = 320 × 320, in-plane resolution = 0.8 × 0.8 mm^2^, slice thickness = 0.8 mm and sagittal slices = 208.

High spatiotemporal resolution fMRI scans are obtained by using a multislice single-shot gradient echo planar imaging sequence: 488 volumes, repetition time = 800 ms, echo time = 37 ms, flip angle = 52°, field of view = 208 × 208 mm^2^, sagittal slices = 72 without slice gaps and matrix size = 2 × 2 × 2 mm^3^. The subjects are instructed to close their eyes but remain awake during the scanning.

### Data pre-processing

The Data Processing Assistant for Resting-State fMRI^49^ (DPARSF) in MATLAB (Mathworks, R2020b), which is a standardized and publically available toolbox, is implemented to pre-process the fMRI data. Each fMRI datum undergoes exclusion of the first eight volumes, head motion correction (Friston-24 model), nuisance covariate regression, spatial normalization to the Montreal Neurological Institute space based on T_1_ for co-registration with Diffeomorphic Anatomical Registration Through Exponentiated Lie Algebra (DARTEL) algorithm,^50^ temporal filtering (0.01 ≤ *f* ≤ 0.1 Hz) and spatial smoothing (full width at half maximum, 4 × 4 × 4). Specifically, the nuisance covariates include head motion, white matter signals and CSF signals.

Note that the data sets for internal validations and external testing are pre-processed independently so that the extractions of dFCN are not inter-correlated upon the application of DARTEL.

### Multiscale dynamic FC

**Figure 1:**
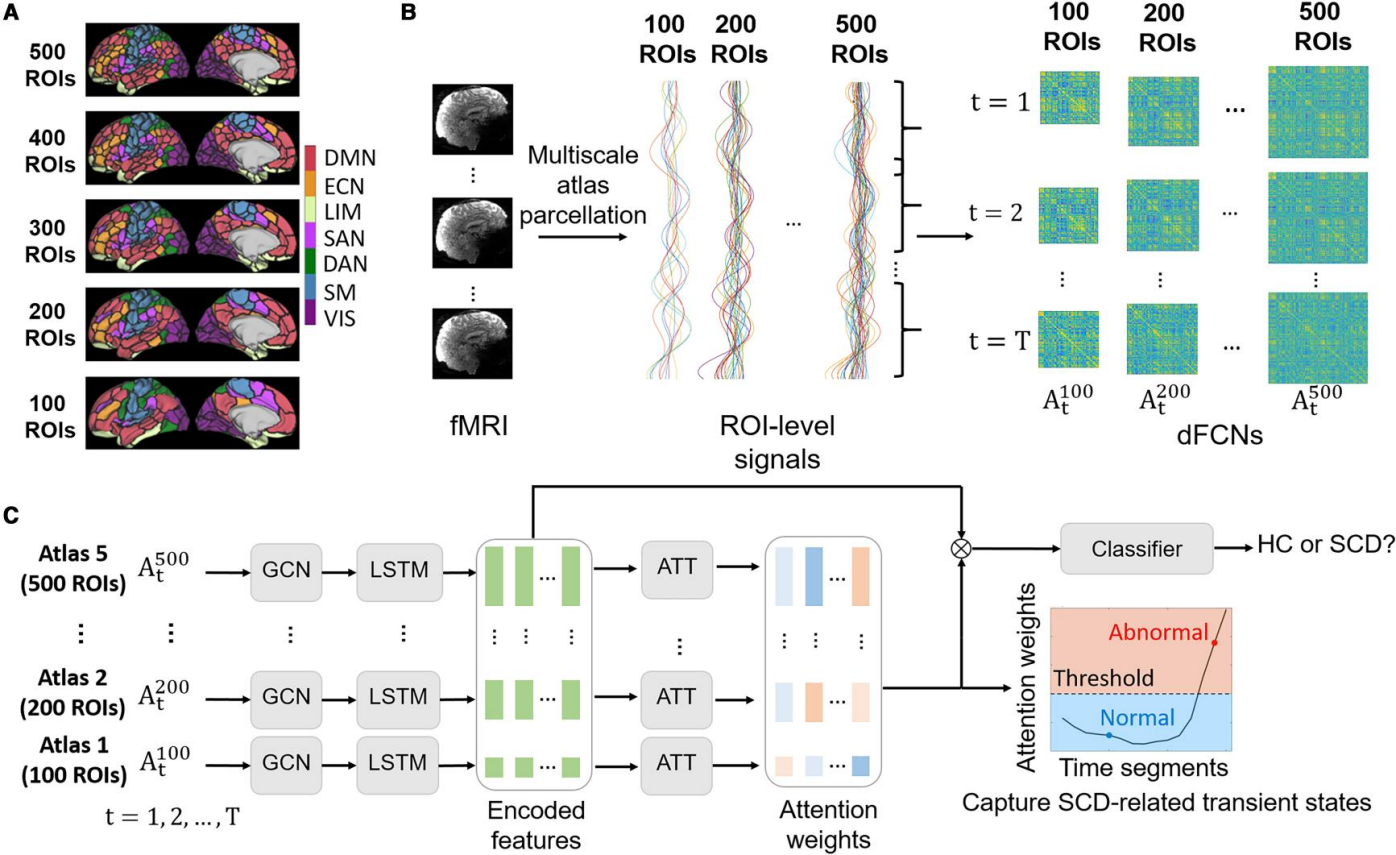
**The workflows of the proposed analysis.** (**A**) The multiscale parcellation atlases. The acronyms of RSNs shown beside the colour bar include default mode network (DMN), executive control network (ECN), limbic network (LIM), salience attention network (SAN), dorsal attention network (DAN), somatomotor network (SM) and visual network (VIS). (**B**) The extraction of multiscale dFCNs from individual fMRI data. (**C**) The proposed deep learning architecture to simultaneously identify SCD and capture SCD-related transient dFCN states.

Based on Schaefer's multiscale brain parcellation atlases,^51^ the multiscale dFCNs are constructed. Schaefer's atlases are generated with local and global similarity-based clustering on the FC profiles from each voxel (or vertex), using 1489 subjects. A tuneable resolution parameter is altered and thus leads to multiscale parcellations [from 100 regions of interests (ROIs) to 1000 ROIs]. Importantly, along the scales changing, the structures of the seven resting-state networks (RSNs) reported by Thomas Yeo *et al*.^52^ are largely preserved. Here, we restrict our analysis to the atlases with scales ranging from 100 to 500 ROIs (Fig. 1A).

Based on Schaefer's parcellations, for each subject, we extract the ROI-wise averaged fMRI signals and construct the dFCNs to characterize temporal variations of the brain's internal functional states (Fig. 1B). Specifically, a sliding window with a length of 48 s and a stride of 16 s is applied and separates the ROI signals into *T* = 22 time segments. Within each time segment, an FC network (a dFCN state) is built by computing pairwise Pearson correlations as edges among the ROI signals. When using an atlas with *R* ROIs (name as the scale, *R* = 100, 200, 300, 400 or 500), the computation leads to a dFCN with dimension R×R×T for each subject. The dFCN state matrix at the segment *t* is denoted as $A_{t}^{R}$, t=1,2,…,T.

### Deep learning architectures

Our deep learning algorithm processing single-scale dFCN consists of three main modules, i.e. graph convolutional neural network (GCN), long short-term memory (LSTM) neural network and attention mechanism (ATT; Fig. 1C). The whole method is then shortly named as ‘G-L-A’. The implementations are done using Pytorch^53^ with programming in Python.

#### GCN

The GCN is an advanced graph deep learning method to sensitively extract features for graph data.^54,55^ Briefly, it aggregates the nodal and topological features via the graph Laplacian and then estimates kernels on the features for selection. During operations, for a given scale *R*, the dFCN state matrix *A_t_* and nodal features $h_{t}$ at *t* undergo the graph convolution operation as follows:


(1) $$\begin{array}{c} h_{t}^{\mathrm{GC}}=\mathrm{GC}(A_{t},h_{t}\;)=\sigma (A_{t}h_{t}W^{R}) \end{array},$$

where the $W^{R}$ is the estimated kernel weight matrix specific for dFCNs at scale R and σ(⋅) is a non-linear rectified linear unit (ReLU) activation function. The $h_{t}$ is set to R×R identity matrix at any *t* and *R*, and therefore, the $h_{t}^{\mathrm{GC}}$ only contains the topological feature of the dFCN state. Note that all dFCN states from the same subject share the same GCN.

#### LSTM neural network

The GCN processed dFCN states at each *t* independently. An LSTM architecture^56^ associated with the GCN at scale *R* further captures the temporal correlations in the dFCN states. The basic recurrent neural network abstracts the ‘hidden state’ of the current input based on the ‘short-term memory’ of several previous hidden states. The LSTM further introduced a time-varying ‘cell state’ $C_{t}$, which is selectively updated by the ‘forgetting phase’ and ‘updating phase’, to maintain the ‘long-term memory’ inside the neural network. At time *t*, in the forgetting phase, a forgetting rate $f_{t}$ was determined depending on the input ( $h_{t}^{\mathrm{GC}}$ from GCN) and the hidden state from the previous time step $h_{t-1}^{\mathrm{LSTM}}$ (from LSTM itself), as follows:


(2) $$\begin{array}{c} \;f_{t}=\sigma (W_{f}h_{t}^{\mathrm{GC}}+U_{f}h_{t-1}^{\mathrm{LSTM}}+b_{f})\; \end{array}.$$

Then in the updating phase, the $h_{t}^{\mathrm{GC}}$ and $h_{t-1}^{\mathrm{LSTM}}$ are combined to set the updating rate $u_{t}$ and context to be memorized ${\tilde{C}}_{t}$ to generate new cell state $C_{t}$, as follows.


(3) $$\begin{array}{c} u_{t}=\sigma (W_{u}h_{t}^{\mathrm{GC}}+U_{u}h_{t-1}^{\mathrm{LSTM}}+b_{u})\; \end{array},$$

(4) $$\begin{array}{c} {\tilde{C}}_{t}=\tanh\;(W_{c}h_{t}^{\mathrm{GC}}+U_{c}h_{t-1}^{\mathrm{LSTM}}+b_{c})\; \end{array},$$

(5) $$\begin{array}{c} C_{t}=f_{t}\times C_{t-1}+u_{t}\times {\tilde{C}}_{t} \end{array}.$$

Finally, the new hidden state $h_{t}^{\mathrm{LSTM}}$ is generated based on $h_{t}^{\mathrm{GC}}$, $h_{t-1}^{\mathrm{LSTM}}$ and $C_{t}$, with


(6) $$\begin{array}{c} o_{t}=\sigma (W_{o}h_{t}^{\mathrm{GC}}+U_{o}h_{t-1}^{\mathrm{LSTM}}+b_{o})\; \end{array},$$

(7) $$\begin{array}{c} h_{t}^{\mathrm{LSTM}}=o_{t}\times \tanh\;(C_{t}) \end{array}.$$

Note that the sum (+) and multiplications (×) are element-wise operations and σ(⋅) is again the ReLU activation function. The initial hidden state $h_{0}^{\mathrm{LSTM}}$ and cell state $C_{0}$ are set to an all-zero matrix.

After the processing of the whole sequence, the LSTM will output the encoded feature for each dFCN state $A_{t}^{R}$ as the hidden state $h_{t}^{\mathrm{LSTM}}$ with consideration of the temporal correlations to the past. Attributing to the recurrent architecture of LSTM, the parameters are jointly trained by all dFCN states from the same subject.

#### ATT

To select the SCD-related states in dFCNs, we apply an ATT^57^ to the outputs from the LSTM. The ‘attention weights’ from this computation are used as a measure of the correlation to SCD on the given dFCN state.

The ATT learns to weigh inputs based on their features and uses these weights for an integration of the inputs. Formally, the temporal features $h_{t}^{\mathrm{LSTM}}$ are dynamically assigned with a weight α(t) and averaged to obtain the weighted averaged feature $h^{\mathrm{ATT}}$, with


(8) $$\begin{array}{c} h^{\mathrm{ATT}}=\overset{T}{\underset{t=1}{\sum }}\;\alpha (t)h_{t}^{\mathrm{LSTM}} \end{array},$$

and α(t) is computed depending on $h_{t}^{\mathrm{LSTM}}$,


(9) $$\begin{array}{c} \alpha (t)=\frac{\exp\;\{W\tanh\;(Vh_{t}^{\mathrm{LSTM}})\}}{\sum_{t=1}^{T}\;\exp\;\{W\tanh\;(Vh_{t}^{\mathrm{LSTM}})\}} \end{array},$$

where W and **V** are parameters to be learned in the ‘attention’ module. Also, note that α(t) is yielded by using a softmax normalization from $W\tanh(Vh_{t}^{\mathrm{LSTM}})$ within a given subject and thus not comparable. We thus use the original amplitude of the unnormalized attention weight $W\tanh(Vh_{t}^{\mathrm{LSTM}})$ to evaluate the abnormality relating to SCD at each dFCN state and monitor the transitions between SCD-related and normal states.

The integrated feature $h^{\mathrm{ATT}}$ is finally forwarded into the one fully connected layer with softmax function for generating diagnosis. All dFCN states from the same subject also utilize the same ‘ATT’ module.

#### Multiscale fusion

We use three methods to perform classifications based on multiscale dFCNs and the previously established G-L-A model at each scale. The parameters in the G-L-A model are frozen during the fusion process.

Firstly, the ‘majority voting’ is implemented to fuse the decisions from the single-scale–based G-L-A models established at the five scales. The predictions from G-L-As are counted as votes. The prediction probability is estimated as the ratio between the number of positive votes and the number of voters (i.e. five models). The final decision is made to the class with the prediction probability being higher than 0.5.

Secondly, the fuse of the decisions is also performed by ‘weighted voting’. In contrast to majority voting equally considering different decisions, the predictions as a probability from the G-L-As are differently treated and combined by weights estimated via another training process.

Thirdly, we fuse the extracted features by each G-L-A, i.e. ‘feature fusion’, rather than integrate different models at the final decision-making stage. The features after the attention process $h^{\mathrm{ATT}}$ from multiple G-L-As are concatenated and processed by two additional fully connected layers with batch normalizations and a softmax function for producing diagnostic probabilities.

Note all multiscale-based methods only conduct fusion based on pre-trained single-scale–based models without altering the attention scores for dFCNs at each scale, and thus, the analysis on their attention weights could be equivalent. Thereby during the analysis of attention, we employ the attention on the dFCN states from the single-scale–based model.

#### Implementation

The proposed G-L-A model is implemented using PyTorch and trained for 100 epochs, using a learning rate = 0.01 and a batch size = 30. For weighted voting and feature concatenation, the models are further trained for 10 epochs, with a learning rate = 0.001 and a batch size = 30. Adam is used as an optimizer with a weight decay = 0.001 to avoid overfitting. The hyper-parameters are optimized using one round of random train–test split before the formal cross-validation. Firstly, based on evidence from training and validating loss, we find the ranges of hyper-parameters, where the model exhibits no obvious overfitting or underfitting. Within the ranges, a grid search is performed. The optimal hyper-parameters are then determined by the set of values obtaining the highest performance during the search. To address the sample imbalance problem, we apply a weighted cross-entropy as the loss function, where the weights are adaptively set to the inverse of the sample ratio in the training set. The training is accelerated by one Nvidia GTX 3080 GPU.

### Validation scheme

In internal validation data sets, 5-fold cross-validation is carried out to evaluate all the above-mentioned models. The data set is randomly separated into five folds with equal sample sizes. Four of the folds are used for model training and the remaining one for testing. The roles of the folds switch five times so that each of the folds is used for testing once. We additionally include two conventional methods to be compared with our proposed method: (i) GCNs (denoted as ‘G’ in the data presentation) based on single-scale static FCNs: the feature of FCN is extracted by a one-layer GCN and processed by two fully connected layers for the diagnosis; and (ii) GCN-LSTM methods based on single-scale dFCNs: this method has a similar architecture as our G-L-A method but without attention (thus termed as ‘G-L’). Further, the single-scale–based method will be compared with the multiscale-based methods to highlight the effectiveness of multiscale dFCN analysis. The results of the internal validations will be utilized to select out optimal models for external testing.

During this process, the data in the external validation data set have not been collected. Then the optimal models selected in the internal validations are fixed and directly applied to the external data sets. The predictions on the external data sets are generated by the majority voting among the five models from cross-validations (the prediction probability is estimated as above). During the model building, the technical team (who develops the model) has access to both MRI data and the diagnosis results from the clinical team (who offers diagnosis decisions and not access to the model development) for the internal data set, while in the final validation, only the MRI data are transferred to the technical team. The model generates the predictions for the subjects in the external data set, which are passed to the clinical team for assessment. The testing process has been done only once throughout the study.

The model performances are quantified by four metrics, i.e. area under the receiver operating characteristic curve (AUROC), area under the precision–recall curve (AUPRC), sensitivity (SEN) and specificity (SPE). The former two metrics offer overall model evaluation under different decision-making probability thresholds and are relatively less influenced by the class imbalance. They are thus used as key metrics for model selections. The latter two provide detailed prediction performance within positive and negative classes, which detects whether an extreme bias to one class exists. We do not apply accuracy as one of the main metrics as the class imbalance induces non-normalized and misleading results. In the internal validation, the mean and standard deviation of the four metrics are reported [in the texts, we report the confidence intervals (CIs) where possible]. For the external validation, we assess the metrics together with their corresponding significances by permutation test. One thousand permutations are conducted to randomize the ground-truth labels, and the performance metrics are re-calculated. The significance (*P*-value) is then yielded as the probability of achieving the metrics larger than that yielded by the model during the permutations.

### Network-level SCD-related dFCN state characterization

Another advantage of our proposed method is the interpretability empowered by the ATT. To illustrate this point, unnormalized attention weights from models built in different folds are first normalized as *Z*-scores and then averaged. A threshold in the attention weight can result in identifications of normal and SCD-related dFCN states (see illustrations in Supplementary Fig. 1).

To characterize the global alternations in SCD-related dFCN states besides local aberrant connections, we compute two network-level metrics based on the network configurations of the dFCN states, namely the modularity and global efficiency. The two metrics are calculated using functions ‘modularity_und’ and ‘efficiency_wei’ from the MATLAB-based toolbox ‘Brain Connectivity Toolbox’ (https://sites.google.com/site/bctnet/home). Before computing the metrics, we consider the FCs with strength above a positive threshold in the dFCN states. All values above the threshold are kept while those below the threshold are set to zero. This is to avoid negative values in the modularity and global efficiency, which causes difficulty in interpretations (Supplementary Fig. 6). Results using threshold = 0 on the FCs are provided in the main texts, and the results under other thresholds are consistent with the presented results. Results under no threshold and other positive thresholds can be found in Supplementary Fig. 6.

#### Modularity

The modular structure is suggested to be essential for the information processing of the brain.^58,59^ The degree to which the brain network is subdivided into clearly delineated communities can be quantified by modularity.^60^ In operations, modularity is defined as the fraction of edges that fall within pre-defined modules, minus the expected number of edges within the modules estimated by randomly rewiring the given network while keeping the nodal degree distribution. Newman's spectral community detection algorithm is used to optimally define the module separations for a given FC structure.^61^

#### Network global efficiency

The global efficiency is the averaged reciprocal of the shortest path length in the network.^62^ It has been shown as a decent quantity for the efficiency of the information processing in the brain functional network and was linked to cognitive abilities.^63^

### Statistical analyses

The differences in gender between groups are tested using $\chi^{2}$ tests with ‘chi2test’ in MATLAB, while the differences in age, education and clinical cognition scores are tested using two-sided two-sample *t*-tests or Whitney–Mann's U-test with ‘ttest2’ and ‘ranksum’ in MATLAB. The model performances in internal validations are compared with one-sided paired *t*-tests (‘ttest’ in MATLAB).

To identify the difference between normal and SCD-related states in dFCN strength and network-level metrics, Whitney–Mann's U-test is performed. When comparing the difference between normal and SCD-related states in terms of dFCN variance, the Levene test is performed using built-in functions ‘vartestn’ in MATLAB (R2020b, Mathworks Inc., USA). To associate the dynamical properties in attention fluctuations and the clinical psychometric scores, Spearman’s correlation is calculated and the corresponding significance is generated by 1000 times of bootstrapping. Under multiple comparisons, the raw *P*-values are corrected by the Bonferroni correction or false discovery rate (FDR) correction with MATLAB.

## Results

### Demographics and clinical characteristics

Table 1 offers the demographic and clinical characteristics of the enrolled subjects in the internal and external validation data sets. In the internal validation data set, no significant differences (at *P* < 0.05 level) are observed in gender, age, education years, MMSE, MoCA-B, AVLT-FDR, AVLT-R, AFT, BNT, STT-A or STT-B among the two groups. There are significant differences in anxiety and depression, measured by HAMD, HAMA and IDS-SR. For the external testing data set, there is no significant difference.

### Abnormality in multiscale dFCNs is robustly identified in SCD

We build and validate our proposed graph deep learning algorithms based on an internal data set and an external data set, respectively (see ‘Materials and methods’).

#### Retrospective internal validation

The results in the internal validation data set are depicted in Table 2. For single-scale–based methods, we use the results from the 500-ROI scale as representatives and offer the results from other scales in Supplementary Table 1. Among these results, we observe that the GCNs based on static FCN generally obtain the lowest AUROCs and AUPRCs than the dFCN-based methods. The G-L method achieves higher AUROCs and AUPRCs than GCNs, but the AUROCs and AUPRCs remain lower than the G-L-A methods. This comparison highlights the advantage of considering dFCN rather than static FCN and capturing the SCD-related temporal states using attention. We further integrate the single-scale–based models for a joint analysis. The multiscale fusion–based methods generally obtain significantly higher AUROCs than single-scale–based models, which demonstrate the cruciality of multiscale dFCN investigations. The weighted voting–based method achieves AUROCs of 0.807 ± 0.046 [CI = (0.744, 0.870)] and AUPRCs of 0.857 ± 0.046 [CI = (0.793, 0.921)], which are higher than all competing models. However, as there is no significant difference between all multiscale fusion–based methods in the results from the internal validation, we opt to select all multiscale fusion–based methods to be tested in the prospective external testing for further comparison.

**Table 2:** Performance data in internal validations based on different methods

Methods	AUROC	AUPRC	SEN (%)	SPE (%)
500-ROI G	0.631 ± 0.059**	0.753 ± 0.072*	61.3 ± 3.2*	64.8 ± 11.4
500-ROI G-L	0.683 ± 0.077*	0.779 ± 0.092	**77.4** ± 4.6	61.8 ± 10.4
500-ROI G-L-A	0.742 ± 0.041*	0.828 ± 0.018	75.1 ± 10.3	63.8 ± 10.7
Feature fusion	0.784 ± 0.036	0.845 ± 0.037	75.6 ± 9.0	**70.2** ± 3.8
Weighted voting	0.769 ± 0.035	0.850 ± 0.047	72.3 ± 10.8	69.4 ± 11.1
Majority voting	**0.807** ± 0.046	**0.857** ± 0.046	75.3 ± 8.8	69.2 ± 8.0

Bold highlights the top performance. *The ‘majority voting’ method exhibits significantly higher metrics than the indicated method at a level of *P* < 0.05 using one-sided paired *t*-tests. **P < 0.01.

#### Prospective external testing

In the external testing, three multiscale-based methods are tested. The five models from the internal validations are integrated to generate one set of predictions on the external data. In Table 3, the predictions from the integrated majority voting–based models are robustly high and significantly above the chance level (AUROC = 0.707, *P* = 0.007, AUPRC = 0.801, *P* = 0.024). Compared with other methods, in terms of AUROC and AUPRC, the advantage of the majority voting–based method is preserved. In addition, it is noted that all the metrics from the majority voting–based method are significantly higher than the chance level. We also investigated the prediction performances of individual models from the internal cross-validation to achieve an uncertainty estimation on the external data (Supplementary Table 2). Overall, applying the five different models independently in the testing data results in AUROCs of 0.616 ± 4.2 [CI = (0.558, 0.673)] and AUPRCs of 0.747 ± 0.029 [CI = (0.706, 0.787)].

**Table 3:** Performance data in external validations based on different methods

Methods	AUROC	*P*	AUPRC	*P*	SEN (%)	*P*	SPE (%)	*P*
Feature fusion	0.668	0.056	0.779	0.167	0.769**	0.004	0.583**	0.003
Weighted voting	0.649	0.081	0.773	0.201	0.769**	0.004	0.583**	0.005
Majority voting	0.707**	0.007	0.801*	0.024	0.885***	<0.001	0.667***	<0.001

Bold highlights the top performance. *The metrics are significantly higher than the chance level at a level of *P* < 0.05 using permutation tests. **P < 0.01. ***P < 0.001.

These results demonstrate that SCD-related abnormalities in multiscale dFCNs can jointly provide comprehensive evidence for better identification of SCD. In addition, these identified abnormalities by our graph learning methods are robust and effective enough to support accurate separations between HC and SCD subjects in the prospective external testing data set.

### Multiscale characterizations for SCD-related states in link and network levels

**Figure 2:**
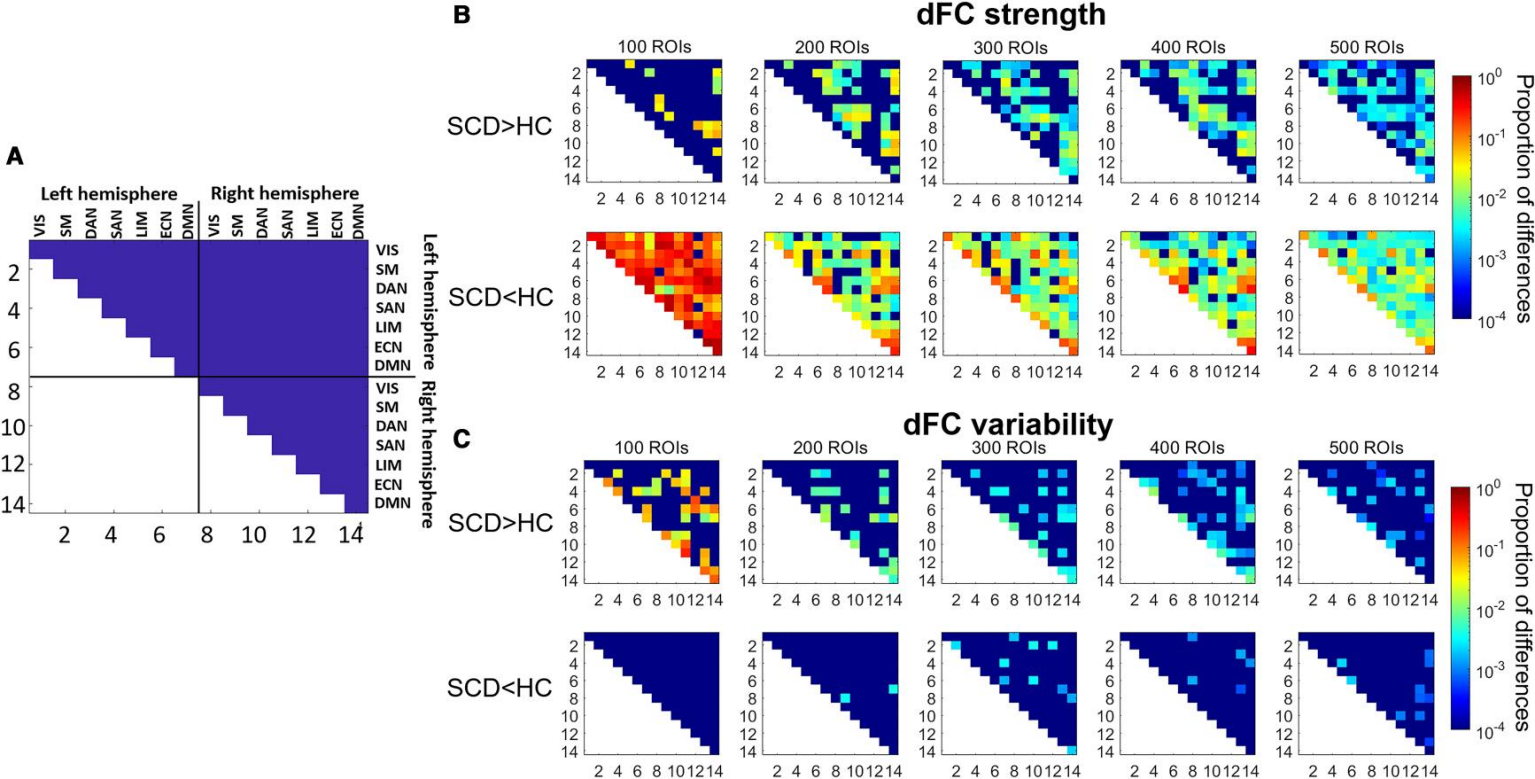
**The proportions of the significantly altered dFCs within different RSNs across spatial scales, in terms of dFC strength and variability.** (**A**) The demonstration of the layout of the presentation of the results. The acronyms of RSNs are the same as Fig. 1A. (**B**) Detected differences in terms of dFC strength. (**C**) Detected differences in terms of dFC variability. The significant increase and decrease are respectively identified. We used one-sided Whitney–Mann's U-tests for strengths and one-sided Levene’s tests for variabilities, at *P* < 0.0001 level after Bonferroni corrections. The percentage of significantly different dFCs within the RSN is then computed and shown in logarithmic scale. The lower triangle parts of the FC matrix are neglected since the matrix is symmetric (shown as white).

**Figure 3:**
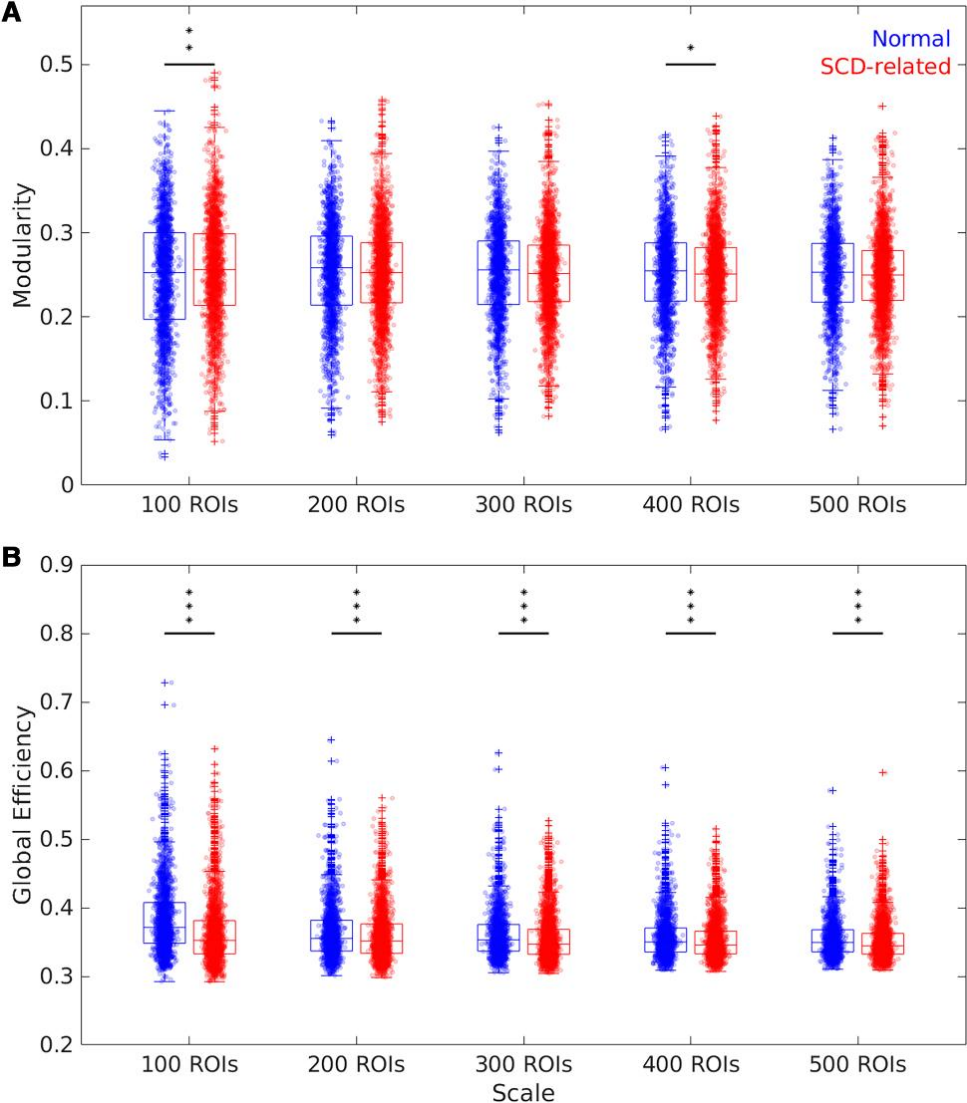
**The distributions of modularity and global efficiency of normal and SCD-related states in multiple scales.** (**A**) The data distributions of modularity. (**B**) The data distributions of global efficiency. A two-sided Whitney–Mann U-test was applied. *P < 0.05. **P < 0.01. ***P < 0.001. *P*-values were uncorrected. For modularity, at 100 ROIs, *P* = 0.0032, the normal (*Z*) statistics = −2.95; at 400 ROIs, *P* = 0.0385, *Z* = 2.07. For global efficiency, 100: *P* = 2.1 × 10^−46^, *Z* = 14.30; 200: *P* = 9.82 × 10^−6^, *Z* = 4.42; 300, *P* = 7.3 × 10^−11^, *Z* = 6.51; 400, *P* = 1.2 × 10^−6^, *Z* = 4.86; 500, *P* = 1.0 × 10^−8^, *Z* = 5.73. Results using threshold = 0 on the FCs are shown.

**Figure 4:**
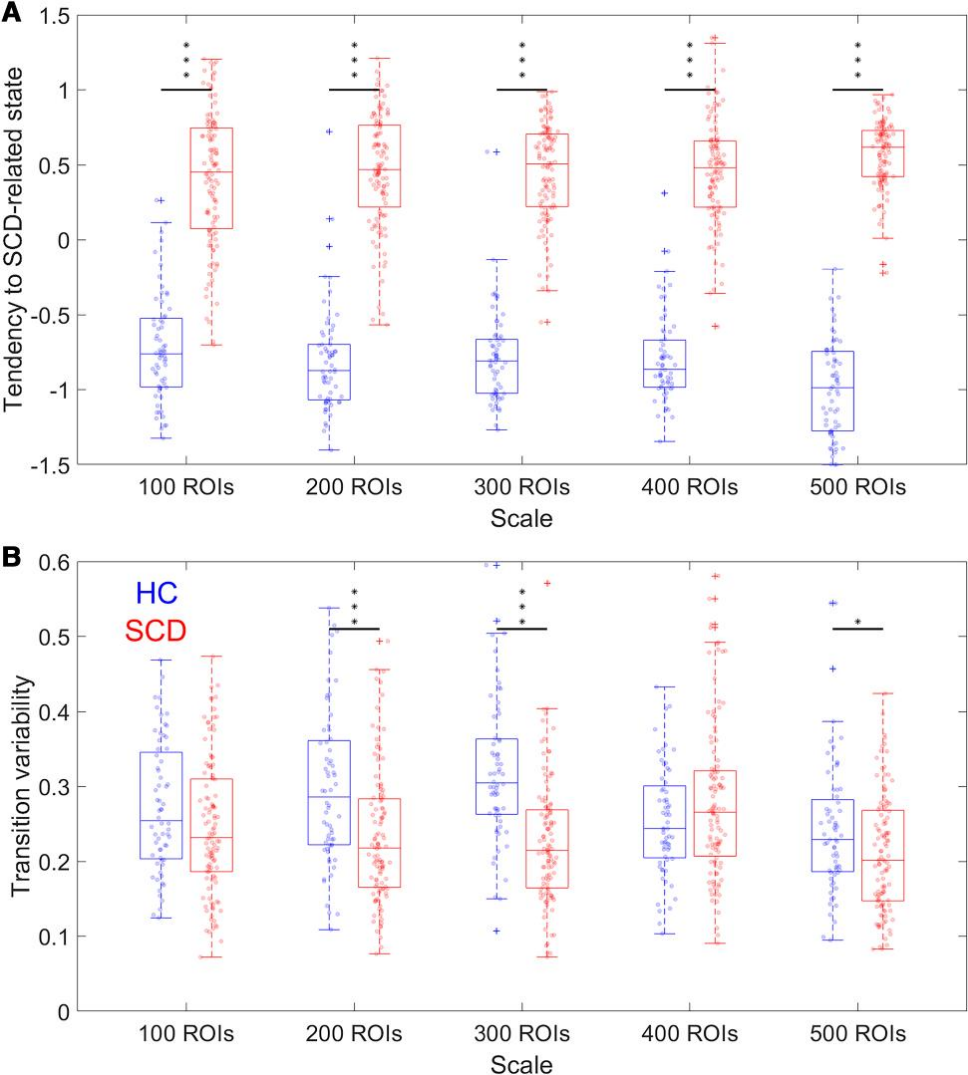
**State transition properties capture inter-group.** The data distributions of the tendency to SCD-related state (**A**) and transition variability (**B**) in HC and SCD groups at multiple scales. *P*-values are estimated with two-sided Whitney–Mann's U-tests and not FDR corrected. *P < 0.05. ***P < 0.001. In **A**, 100: *P* = 4.31 × 10^−25^, *Z* = −10.35; 200: *P* = 3.00 × 10^−26^, *Z* = −10.60; 300: *P* = 4.20 × 10^−27^, *Z* = −10.78; 400: *P* = 2.30 10^−27^, *Z* = −10.84; 500: *P* = 3.00 × 10^−28^, *Z* = −11.02. In **B**, 200: *P* = 2.18 × 10^−5^, *Z* = 4.25; 300: *P* = 1.05 × 10^−10^, *Z* = 6.46; 500: *P* = 0.0219, *Z* = 2.29.

The ATT implemented inside our model enables the exploration of the characteristics of SCD-related dFCN states and the transitions between normal and SCD-related states (see ‘Materials and methods’), which would deepen the understanding of neurological alternations underlying SCD. In the following analyses (Figs 2–4), we investigate these aspects using the retrospective internal validation data.

In Supplementary Fig. 1, the averaged normalized attentions are first visualized. The attention weights on the dFCN states from the SCD subjects generally separate from the dFCN states from HC subjects. The mean attention level (i.e. zero, after *Z*-score transformation) could be roughly used as an intuitive threshold to separate the normal and SCD-related dFCN states. However, there is a noticeable overlap between the attention distributions in HC and SCD groups (Supplementary Fig. 1). The SCD-related states can emerge in HC subjects while normal states can appear in SCD subjects (see cases in Supplementary Fig. 2). Despite the limited classification capacity of the model, this phenomenon could indicate the state transitions between normal and SCD-related dFCN states in both HC and SCD subjects.

The identified SCD-related dFCN states are systematically characterized at link and network levels. Firstly, we group normal and SCD-related states indicated by the model and performance state-wise comparison in terms of the dFCN strength and variability. The significantly different dFCs (provided in Supplementary Figs 3 and 4) are counted and converted to proportion according to their belongings to different RSNs across different scales (Fig. 2). The results depict that dFCNs are more frequently showing decreased dFC strength and increased variability in SCD-related states. In addition, in intra-hemisphere dFCs within left DMN, right DMN, right executive control network (ECN) and left dorsal attention network (DAN) and the inter-hemisphere dFCs between left and right DMNs, decreased strengths are consistently exhibited, in all the investigated scales. We additionally perform comparisons among states grouped according to the diagnosis of the subjects (e.g. all states from SCD subjects are regarded as SCD-related states). In Supplementary Fig. 5, under the subject-level grouping, the decreased strength and increased variability of dFCs in SCD can be detected and dFC strength reductions are observed at all scales in DMNs and right ECN.

At the whole network level, we investigate two aspects of the dFCNs under normal and SCD-related states, namely modularity and global efficiency. The results with threshold = 0 (see ‘Materials and methods’) on the FCs are shown in Fig. 3, while the results with other thresholds are offered in Supplementary Fig. 6. The results across different thresholds are largely consistent. At the 100-ROI scale, the SCD-related state exhibits significantly increased modularity than normal states (*P* = 0.0032, FDR-corrected *P* = 0.016). On the contract, at other scales, the SCD-related state shows decreased modularity. At the 400-ROI scale, a significantly decreased modularity is identified (*P* = 0.0385, FDR-corrected *P* = 0.096) in SCD-related states. For global efficiency, SCD-related states show significantly decreased global efficiency than normal states at all investigated scales (100: *P* = 2.1 × 10^−46^, FDR-corrected *P* = 1.1 × 10^−46^; 200: *P* = 9.82 × 10^−6^, FDR-corrected *P* = 9.82 × 10^−6^; 300, *P* = 7.3 × 10^−11^, FDR-corrected *P* = 1.82 × 10^−10^; 400, *P* = 1.2 × 10^−6^, FDR-corrected *P* = 1.46 × 10^−6^; 500, *P* = 1.0 × 10^−8^, FDR-corrected *P* = 1.69 × 10^−8^). In Supplementary Fig. 7, when grouping the state based on the subject-level diagnosis, there is no significant difference observed in modularity while the global efficiencies at all scales are significantly decreased.

### State transition properties reflect group differences and individual differences in clinical psychometrics at different scales

**Figure 5:**
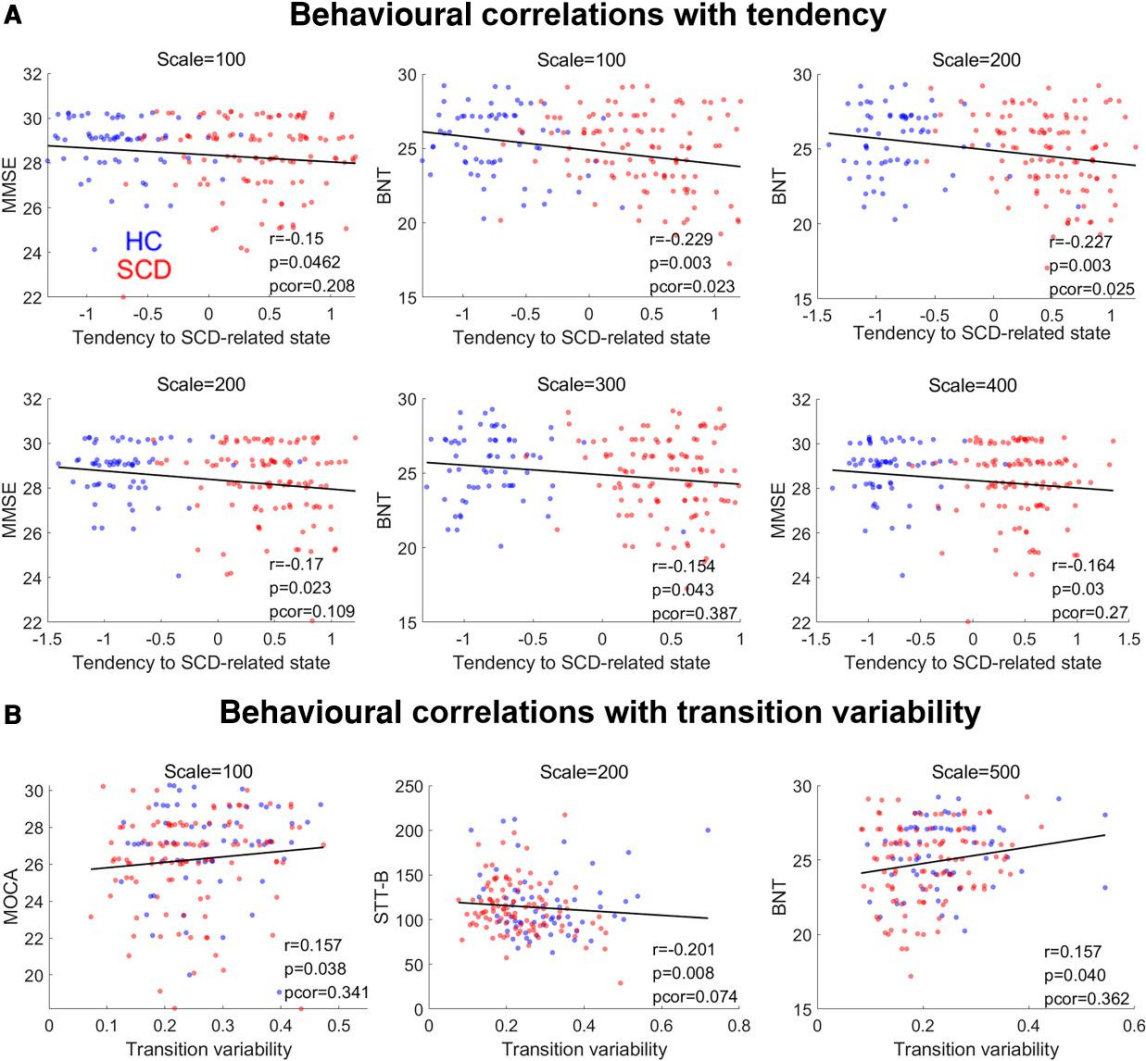
**State transition properties capture inter-individual differences in clinical psychometrics.** Scatter plots for correlations between state transition properties at different scales and clinical psychometrics. **A** is for the tendency to SCD-related state and **B** is for the transition variability. The Spearman correlation *r*, raw *P*-values and FDR-corrected *P*-values (pcor) are depicted at the right bottom of each plot.

The time evolution of attention can reflect the state transitions, and thus, the mean level and variability (standard deviation) correspond to the tendency to stay in an SCD-related state and transition variability during the brain dynamics. We quantify these properties to detect group differences and to investigate their inter-individual correlations to the clinical psychometric scores (Figs. 4 and 5). We expect that a higher tendency in SCD-related state relates to worse cognitive performance, while a high transition variability indicates flexible state transitions and may reflect better cognitive performance. In Fig. 4A, we observe that SCD subjects exhibit a significantly higher tendency to stay in an SCD-related state at all five scales (all *P* < 0.001 after FDR corrections). Consistently, the tendencies to SCD-related state are negatively and significantly associated with MMSE and BNT, reflecting overall cognitive ability and language processing, across individuals at 100- to 400-ROI scales (Fig. 5A). The correlations to BNT at 100- and 200-ROI scales remain significant after FDR correction. Meanwhile, the transition variabilities in SCD population significantly drop at 200-, 300- and 500-ROI scales (Fig. 4B) and are positively associated to MoCA and BNT and negatively correlated to STT-B with significance before correction (Fig. 5B). Since lower STT-B indicates better cognitive ability, these results generally support our hypothesis that the low tendencies in SCD-related state and high transition variability, informed by the attentions, correspond to the better cognitive status in subjects.

## Discussion

### Abnormality in the brain multiscale dynamic functional network as a neurological manifestation of SCD

The nature of SCD is controversial due to the lack of objective biomarkers since being proposed. Recent studies based on brain networks identified a few imaging markers to support SCD as a symptom entity.^11^ Expanding the investigation scope to the multiscale brain functional networks (from single-scale static FCN to multiscale dFCN) and utilizing the sensitive feature identifications of advanced graph learning, our analyses illustrate SCD can be associated with the objective multiscale transient abnormality in the brain functional interactions. Such objective imaging marker is also shown to be reproducible as SCD subjects from a prospectively collected external data set can be sensitively identified. These results could significantly facilitate the systematic studies of SCD for clinical purposes. However, our evidence may still be limited due to the lack of validations on other cohorts, and thus, more systematic testing across multiple centres is needed in future works. In the inter-centre validations, the inconsistencies in scanning protocol and diagnosis criteria are required to be addressed with further methodological developments, which are so far out of the scope of the presented work.

### Insufficient neural information processing in SCD

The state-wise comparison suggests the SCD could be linked to decreased dFC strengths, which could indicate a less efficient information exchange. Consistently, at the network level, the SCD-related states are also featured by the alternations in global efficiency of the transient functional network configurations at all scales. Meanwhile, we witness enhanced modularity in SCD-related states at the coarse spatial scale (e.g. 100-ROI scale) while those at fine scales exhibit undermined modularity (e.g. 400-ROI scale). Since modularity measures the information segregation in the network structure, the SCD may thus related to increased coarse-scale segregation and fine-scale integrations, which may both be due to the dysfunction of long-range connections. Consistently, across the individuals, high dwell times in such SCD-related states, expected to be with low effectiveness in information processing, are associated with low cognitive performances.

### Cognitive maintenance via functional network reconfigurations

Despite the insufficient neural information processing, at the local connection level, we also find increased dFC variabilities in the SCD-related dFCN states. The increased dFC variability may underpin a functional re-organization to build alternative signalling pathways and maintain cognitive functions, as previously suggested in the Scaffolding Theory of Cognition.^24^ In parallel, the high transition variability among the states could be correlated to better cognitive performances across subjects (in the whole group and SCD group), which further supports the benefits of flexible network reconfiguration on cognitive functioning. However, at the group level, SCDs generally exhibit lower state transition variability than the HCs. It could still be understood since the flexible connection reconfigurations may not lead to effective state transitions from SCD-related states to normal states. In SCD subjects, the state transition is restricted within SCD-related states (indicated by a higher tendency to SCD-related states) and thus the transition variability is still reduced when compared to HCs. The effect of maintenance could thus be limited, and the SCD is still a status deviated from the normal cognition.

Interestingly, it is observed that the success of multiscale-based predictions needs the failures of dFCNs at a majority of scales (e.g. the majority voting). This result implies the multiscale dFCN could play a role in cognitive maintenance.^35^ When the dFCNs at a majority of scales remain normal functioning, the information could be successfully routed in the brain hierarchical network and the symptoms of SCD may not be presented.

### Non-memory deficits in SCD

In addition, a large proportion of the dFCs in DMN, ECN and ATT are found to be depressed by SCD, which is consistently observed across multiple spatial scales. Pronounced alternations in the limbic network are not observed, which are featured in the amnestic mild cognitive impairment and Alzheimer's disease–induced dementia. On the other hand, the dwell time in SCD-related state at multiple scales are frequently associated with MMSE and BNT scores, corresponding to global and language abilities, and the transition variabilities among states are linked to the cognitions in global, language and executive function domains. Therefore, both of the dynamical properties of the attention (dFCN state transitions) are not associated with memory-related scores. It may potentially be attributed to the early-stage nature of SCD in the progression to dementia, where objective memory impairment is not presented but cognitive deficits in other domains, such as attention, working memory and executive function, may occur. Theoretically, the cognitive deficits could essentially underlie the subjective experiences of memory decline. A previous conceptual theory proposed by Jessen *et al.*^1^ also indicates that the self-reported memory decline may be attributed to impairments in other cognitive domains. Therefore, the interplays between subjective memory decline and impairments in other cognitive domains could be an interesting topic to be explored under the context of SCDs.

### Dependencies between SCD and Alzheimer's disease

Previous studies highlighted the deficits of DMN in SCD and the progressions in Alzheimer's disease.^3,10,17,18,64,65^ Here, we collect consistent evidence for such deficits and further demonstrate their occurrence across multiple spatial scales in SCD. In Alzheimer's disease and mild cognitive impairment studies, it was also reported that the global efficiency in functional networks is decreased.^66^ In a magnetoencephalography-based Alzheimer's disease study, the modularity of brain functional networks was found to increase in the delta and theta bands (slow temporal scale) while decreasing in the beta and gamma bands (fast temporal scale).^67^ The slow or fast temporal scale could correspond to the coarse or fine spatial scale since they respectively reflect the global or local neural communications.^68,69^ Therefore, the observed SCD-related network-level changes are in the same direction as those induced by Alzheimer's disease. However, the current study could be limited to concluding the relationship between SCD and Alzheimer's disease, because of the lack of longitudinal following.

### Effects of anxiety and depression

In the internal validation data set, significantly higher levels of anxiety and depression SCD are detected in HC, which is in line with previous studies in different cohorts.^23,70-72^ The effect of anxiety and depression may thus be a crucial aspect to be explored to reveal the nature of SCD. However, note the significant difference is not found in the subjects for external validation, where our models can still accurately identify these SCD subjects. Therefore, our results and the corresponding interpretations can be robust to the effects of anxiety and depression.

## Conclusion

In the presented work, we reveal the dysfunctions of the multiscale dynamical brain functional networks can be a reliable neural manifestation of SCD. Firstly, with the advanced graph deep learning methods to multiscale dFCNs, we demonstrate that jointly considering abnormalities from multiscale can reliably and precisely identify subjects under SCD. In addition, we analyse the predictive features in the normal and SCD-related dFCN states and their temporal transitions, revealed by the ATT implemented in our models. We observe that SCD-related dFCN states typically exhibit decreased dFC strengths and increased dFC variability when compared to normal states. The majority of the dFCs within DMN, ECN and DAN exhibit decreased strengths, which can be consistently observed across multiple spatial scales. In addition, SCD-related dFCN states are also featured by the changes in modularity at specific scales and decreased global network efficiency across all scales. Besides, the dynamical properties of the state transitions derived from the attention time series are found to be informative to the clinical psychometrics across subjects.

To conclude, the presented study supports the objective neurological alternation underlying SCD from the perspective of multiscale dynamic brain functional networks. The analysis also deepens the understanding of the neural and cognitive deficits in SCD, and the established methods could pave the way to the clarification of the relationship between SCD and early-stage Alzheimer's disease.

## Supplementary material

Supplementary material is available at *Brain Communications* online.

## Supplementary Material

fcae010_Supplementary_Data

## Data Availability

The data that support the findings of this study are available at Zenodo (DOI: 10.5281/zenodo.10046649). Original data are available from the corresponding author upon reasonable request due to restrictions of the hospital. All the codes and established models in this study are publicly available on GitHub (https://github.com/MianxinLiu/Dysfunctions-of-Multiscale-Dynamic-Brain-Functional-Networks-in-Subjective-Cognitive-Decline).
