# Supplementary material for: Dysfunctions of multiscale dynamic brain functional networks in subjective cognitive decline
Source: Brain Commun. 2024 Jan 16;6(1):fcae010. doi: 10.1093/braincomms/fcae010 (PMC10833653; doi:10.1093/braincomms/fcae010)
Supplement: fcae010_Supplementary_Data [file fcae010_supplementary_data.pdf]

Dysfunctions of Multiscale Dynamic Brain Functional Networks in Subjective Cognitive Decline

Mianxin Liu, Qi Huang, Lin Huang, Shuhua Ren, Liang Cui,

Han Zhang, Yihui Guan, Qihao Guo, Fang Xie, Dinggang Shen

Supplementary Materials

Supplementary Table: 2

Supplementary figures: 7

Supplementary Table 1. Performance data in internal validations based on different methods. *: the "Majority Voting" method exhibits significantly higher metrics than the indicated method at a level of $p<0.05$ using one-sided paired t-tests. **: $p<0.01$. ***: $p<0.001$.

**Methods**	**AUROC**	**AUPRC**	**SEN (%)**	**SPE (%)**
100-ROI G	0.619±0.037**	0.706±0.108*	63.6±5.5*	60.3±10.9
200-ROI G	0.598±0.060**	0.678±0.032**	63.6±13.0	56.0±2.1*
300-ROI G	0.629±0.046**	0.743±0.094*	60.9±7.1*	64.8±8.7
400-ROI G	0.618±0.074**	0.744±0.097*	59.7±5.4*	64.0±9.9
500-ROI G	0.631±0.059**	0.753±0.072*	61.3±3.2*	64.8±11.4
100-ROI G-L	0.682±0.047*	0.803±0.043	62.5±12.5*	**75.1**±14.4
200-ROI G-L	0.633±0.107*	0.738±0.131	73.1±13.6	65.0±6.7
300-ROI G-L	0.635±0.077*	0.751±0.119	69.1±8.7	73.2±14.6
400-ROI G-L	0.684±0.086*	0.775±0.044*	**78.6**±7.2	61.7±5.4
500-ROI G-L	0.683±0.077*	0.779±0.092	77.4±4.6	61.8±10.4
100-ROI G-L-A	0.718±0.026*	0.818±0.099	68.6±7.7*	68.1±15.6
200-ROI G-L-A	0.724±0.042**	0.820±0.049	58.9±4.8**	71.8±4.6
300-ROI G-L-A	0.703±0.047**	0.801±0.067	72.5±6.5	64.4±17.9
400-ROI G-L-A	0.735±0.054*	0.806±0.040*	74.8±11.2	62.8±10.8*
500-ROI G-L-A	0.742±0.041*	0.828±0.018	75.1±10.3	63.8±10.7
Feature fusion	0.784±0.036	0.845±0.037	75.6±9.0	70.2±3.8
Weighted voting	0.769±0.035	0.850±0.047	72.3±10.8	69.4±11.1
Majority voting	**0.807**±0.046	**0.857**±0.046	75.3±8.8	69.2±8.0

Supplementary Table 2. Performance in external testing using a majority-voting model trained on different data splits in internal cross-validation (CV). *: the predictability is significant at a level of $p<0.05$. **: $p<0.01$.

Data split	AUROC	AUPRC	SEN (%)	SPE (%)
Split CV1	0.671	0.769	84.6	58.3
Split CV2	0.590	0.744	0.692	50
Split CV3	0.614	0.754	0.846	25
Split CV4	0.649	0.775	0.692	50
Split CV5	0.554	0.692	0.577	58.3

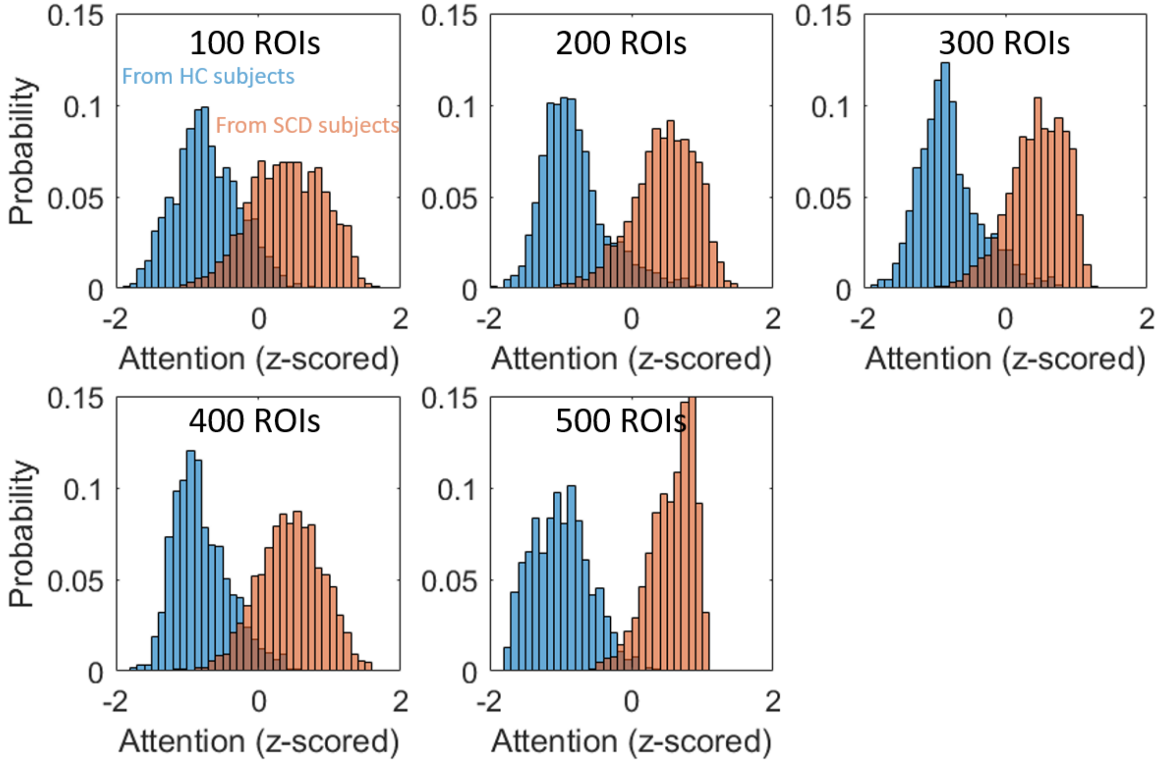

Supplementary Figure 1. The distributions of fold-wise averaged normalized attention in healthy control and SCD subjects. The attention weights indicate a higher correlation to SCD for dFCN states as higher values. The mean attention level (i.e., zero, after z-score transformation) could be roughly used as an intuitive threshold to separate the normal and SCD-related dFCN states. Associated to Fig. 2 in the main text.

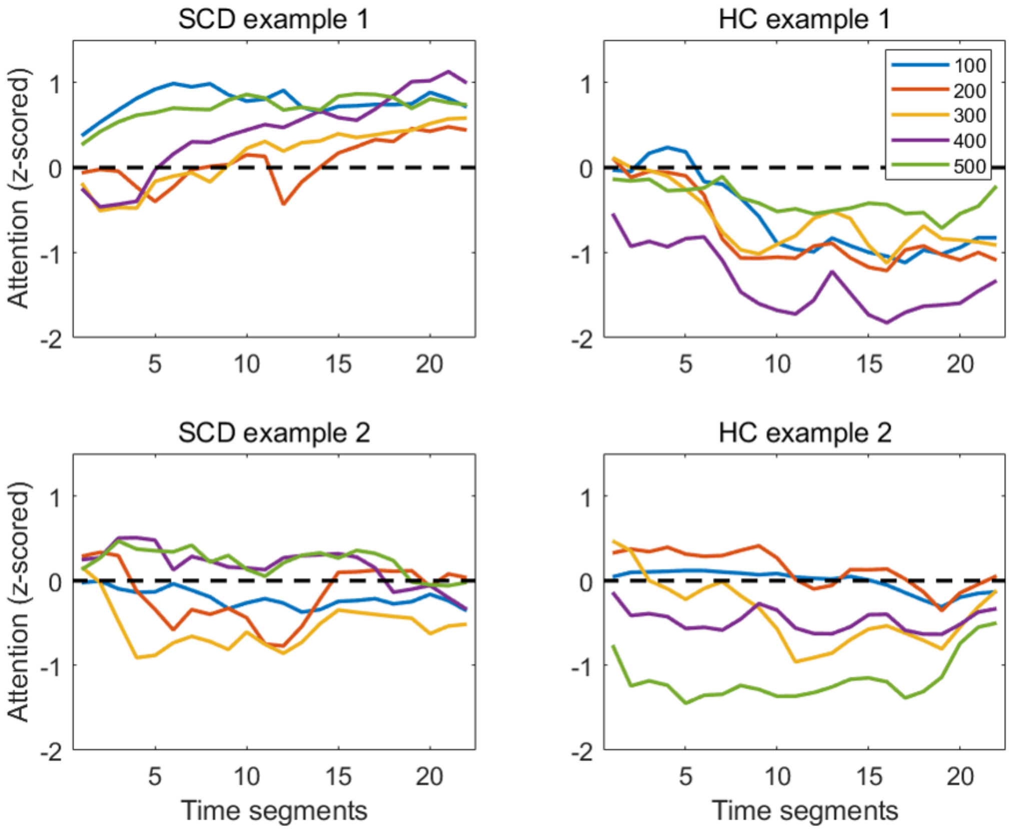

Supplementary Figure 2. The temporal fluctuations of the attentions on dFCN transitions at multiple scales from exemplified participants. Based on Fig. S1, the mean attention level (i.e., zero after the z-score) is used as a guiding threshold to separate the normal and SCD-related states. Associated to Fig. 2 in the main text.

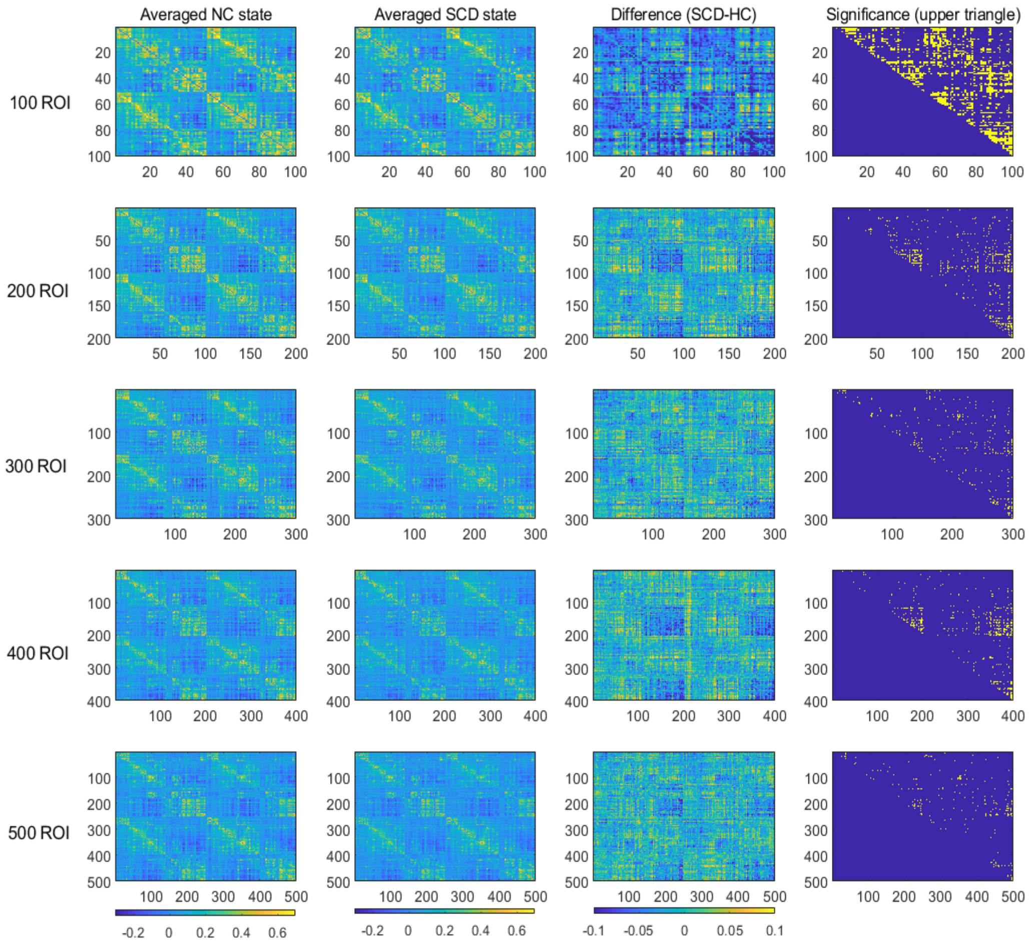

Supplementary Figure 3. State-wise comparison in terms of dFC strength at different scales. Associated to Fig. 2 in the main text.

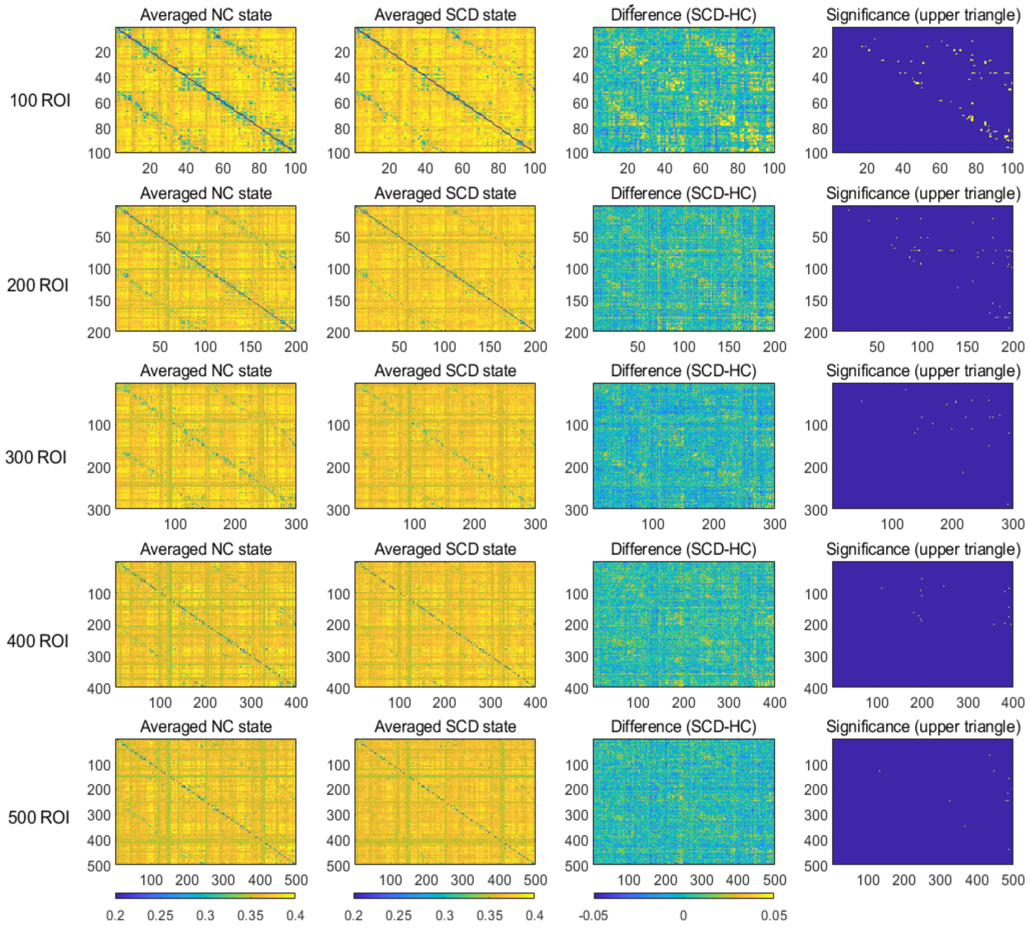

Supplementary Figure 4. State-wise comparison in terms of dFC variability at different scales. Associated to Fig. 2 in the main text.

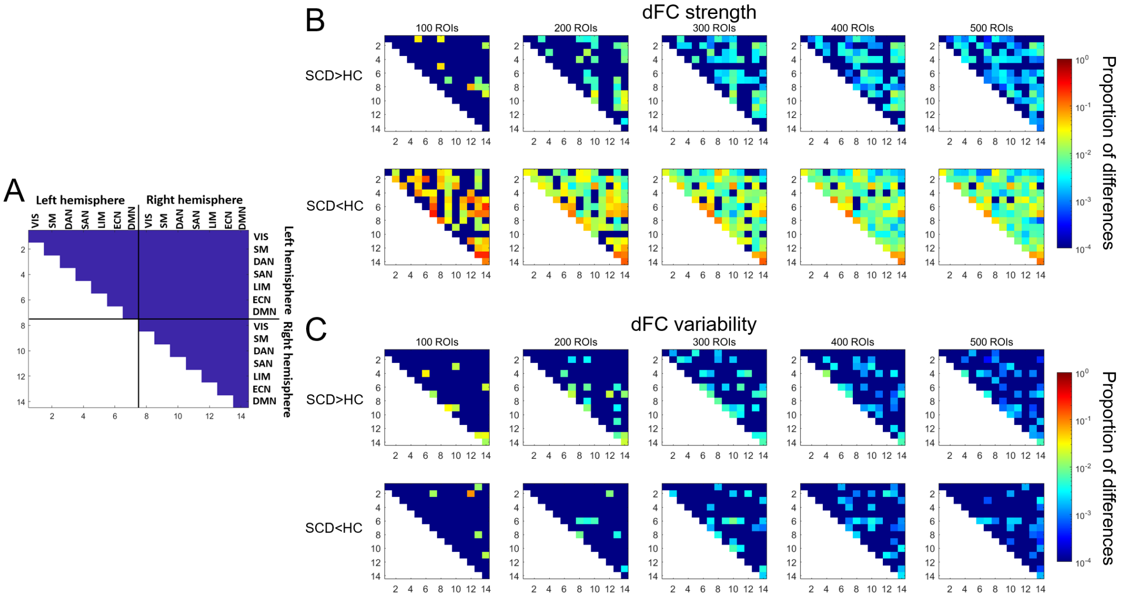

Supplementary Figure 5. The proportions of the significantly altered dFCs within different resting-state networks across spatial scales, in terms of strength and variability, based on participant-level state grouping. Associated to Fig. 2 in the main text.

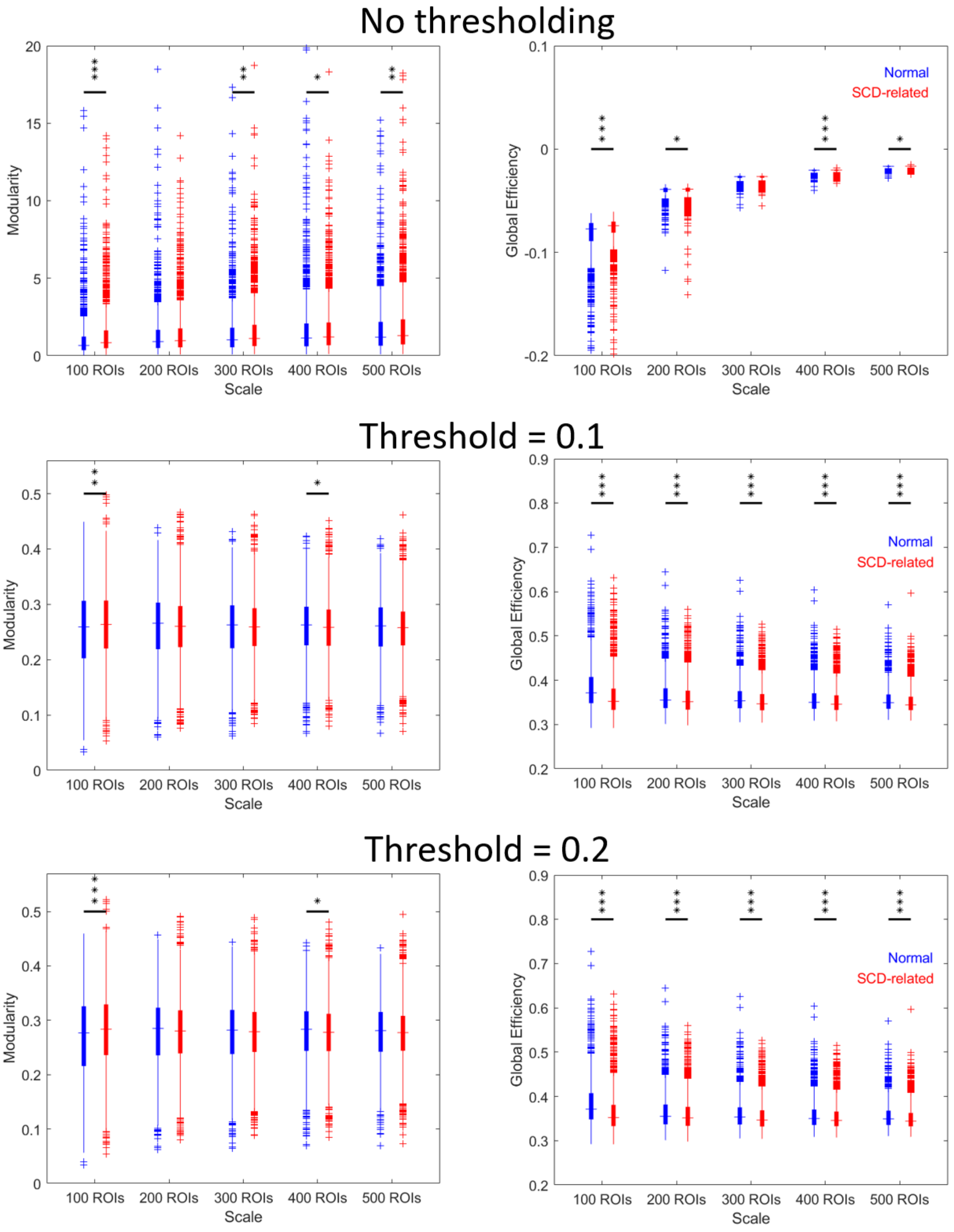

Supplementary Figure 6. The distributions of modularity and global efficiency of normal and SCD-related states in multiple scales, under different thresholds on dFCN. A two-sided Whitney-Mann's U test was applied. *: $p < 0.05$. **: $p < 0.01$. ***: $p < 0.001$. p-values were uncorrected. Associated to Fig. 3 in the main text.

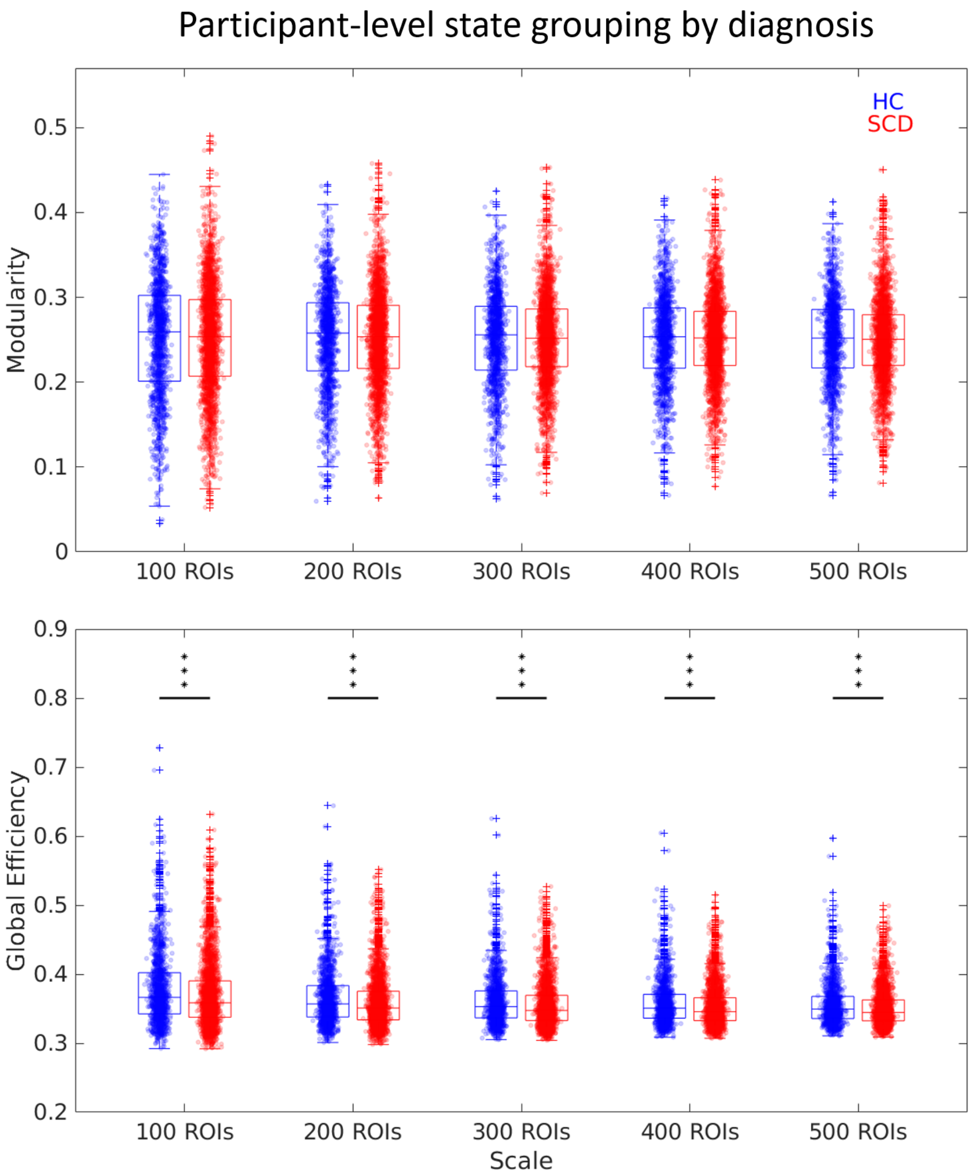

Supplementary Figure 7. The distributions of modularity and global efficiency of normal and SCD-related states in multiple scales based on participant-level state grouping. A two-sided Whitney-Mann's U test was applied. *: p <0.05. **: p <0.01. ***: p <0.001. p-values were uncorrected. Results using threshold = 0 are shown. Associated to Fig. 3 in the main text.
